# Reactive sulfur species disaggregate the SQSTM1/p62-based aggresome-like induced structures *via* the HSP70 induction and prevent parthanatos

**DOI:** 10.1016/j.jbc.2023.104710

**Published:** 2023-04-13

**Authors:** Yutaro Yamada, Takuya Noguchi, Midori Suzuki, Mayuka Yamada, Yusuke Hirata, Atsushi Matsuzawa

**Affiliations:** Laboratory of Health Chemistry, Graduate School of Pharmaceutical Sciences, Tohoku University, Sendai, Japan

**Keywords:** reactive sulfur species, parthanatos, aggresome, p62 (sequestosome 1(SQSTM1)), 70 kDa heat shock protein (Hsp70)

## Abstract

Reactive sulfur species (RSS) have emerged as key regulators of protein quality control. However, the mechanisms by which RSS contribute to cellular processes are not fully understood. In this study, we identified a novel function of RSS in preventing parthanatos, a nonapoptotic form of cell death that is induced by poly (ADP-ribose) polymerase-1 and mediated by the aggresome-like induced structures (ALIS) composed of SQSTM1/p62. We found that sodium tetrasulfide (Na_2_S_4_), a donor of RSS, strongly suppressed oxidative stress-dependent ALIS formation and subsequent parthanatos. On the other hand, the inhibitors of the RSS-producing enzymes, such as 3-mercaptopyruvate sulfurtransferase and cystathionine γ-lyase, clearly enhanced ALIS formation and parthanatos. Interestingly, we found that Na_2_S_4_ activated heat shock factor 1 by promoting its dissociation from heat shock protein 90, leading to accelerated transcription of HSP70. Considering that the genetic deletion of HSP70 allowed the enhanced ALIS formation, these findings suggest that RSS prevent parthanatos by specifically suppressing ALIS formation through induction of HSP70. Taken together, our results demonstrate a novel mechanism by which RSS prevent cell death, as well as a novel physiological role of RSS in contributing to protein quality control through HSP70 induction, which may lead to better understanding of the bioactivity of RSS.

Parthanatos is a form of nonapoptotic programmed cell death governed by poly (ADP-ribose) polymerase-1 (PARP-1) (1). Under physiological conditions, PARP-1 mainly contributes to DNA repair and maintenance of genomic stability through poly-ADP-ribosylation of nuclear proteins (2). However, under pathological conditions, PARP-1 mediates proinflammatory responses and cell death (1, 3, 4). Importantly, pathologic stresses cause overactivation of PARP-1, and thereby poly (ADP-ribose) (PAR) is overproduced and released into the cytoplasm. Cytoplasmic PAR preferentially binds to apoptosis-inducing factor (AIF) and then promotes its nuclear translocation, which triggers DNA fragmentation associated with parthanatos (1). Notably, the overactivation of PARP-1 is observed in the lesions of neurodegenerative diseases, such as Parkinson's disease, Alzheimer's disease, and amyotrophic lateral sclerosis (5, 6, 7). Moreover, parthanatos has been shown to be involved in the neurologic deficits in these neurodegenerative diseases (7, 8, 9). In particular, PAR has been reported to increase the toxicity of the protein aggregates containing α-synuclein or TDP-43, suggesting a mechanistic link between protein aggregates and parthanatos (8, 9).

Oxidative stress causes protein misfolding and then promotes the formation of the protein aggregates that are called aggresome-like induced structures (ALIS) (10). Although the detailed mechanism of the ALIS formation has not yet been elucidated, it has been demonstrated that the autophagy receptor SQSTM1/p62 is a component of ALIS and plays an important role in the ALIS formation (11, 12, 13). Interestingly, p62 is included in the neurodegenerative disease-related aggregates such as Lewy body, Huntington's aggregate, and in the neurofibrillary degeneration in Alzheimer's disease (14, 15, 16, 17, 18). Moreover, oxidative stress promotes the formation of p62-based ALIS that triggers parthanatos (13). Therefore, suppression of the formation of p62-related aggregates may lead to the treatment of neurodegenerative diseases.

The accumulation of reactive oxygen species (ROS) initiates oxidative stress that causes oxidation of cysteine residues in proteins. Recently, the critical roles of reactive sulfur species (RSS), such as cysteine hydropersulfide, that protect proteins from irreversible oxidation are highlighted (19). To date, several enzymes that produce RSS, including 3-mercaptopyruvate sulfurtransferase (3-MST), cystathionine γ-lyase (CSE), cystathionine β-synthase, and cysteinyl-tRNA synthetase , have been identified and turned out to be important in the regulation of redox signaling and the elimination of ROS (20, 21, 22). Moreover, recent evidence has shown that persulfidation (Cys-SSH) protects cysteine residues from its irreversible oxidation that leads to loss of protein function, suggesting that RSS play a pivotal role in cellular protein quality control (19).

Inorganic sodium polysulfides (Na_2_S_n_) can mimic endogenously produced polysulfides and thereby are used as RSS donors in experiments (23). These polysulfides prevent stress-induced apoptosis by activating the NF-E2–related factor 2 signaling pathways or by promoting the sulfhydrylation of sirtuin 1 (24, 25). Moreover, polysulfide suppresses ferroptosis, a form of nonapoptotic cell death caused by lipid peroxidation, through the production of glutathione persulfide (26). Therefore, RSS have been suggested to be inhibitory factors of programmed cell death.

Heat shock proteins (HSPs) are a group of highly conserved chaperone molecules that are induced in response to heat shock and other stresses (27). HSPs refold denatured proteins or promote proteasomal degradation of denatured proteins during various stresses, which contribute to protection from stress-induced cytotoxicity (28, 29). In particular, HSP70 plays a central role in protein quality control and prevents protein aggregation through binding to hydrophobic residues of denatured proteins (30). Since HSP70 also suppresses the aggregation of α-synuclein and amyloid β, HSP70 inducers are expected to be applied to the treatment of neurodegenerative diseases (31). The expression of HSPs is mainly regulated by the transcription factor heat shock factor 1 (HSF1) (27). The transcriptional activity of HSF1 is constantly suppressed by the binding of HSP90, but various stresses disrupt this binding and thereby promote the trimerization and nuclear translocation of HSF1, which allows to exert transcriptional activity of HSF1 (32).

In this study, we found that RSS promote the induction of HSP70 expression at both protein and mRNA levels through the HSF1 activation and thereby suppress parthanatos by preventing the p62-based ALIS formation. Thus, our findings demonstrate a novel RSS-mediated inhibitory mechanism of cell death and a novel signaling mechanism that RSS activate HSF1 through the disruption of the HSP90–HSF1 complex.

## Results

### RSS suppress oxidative stress-induced parthanatos

**Figure 1:**
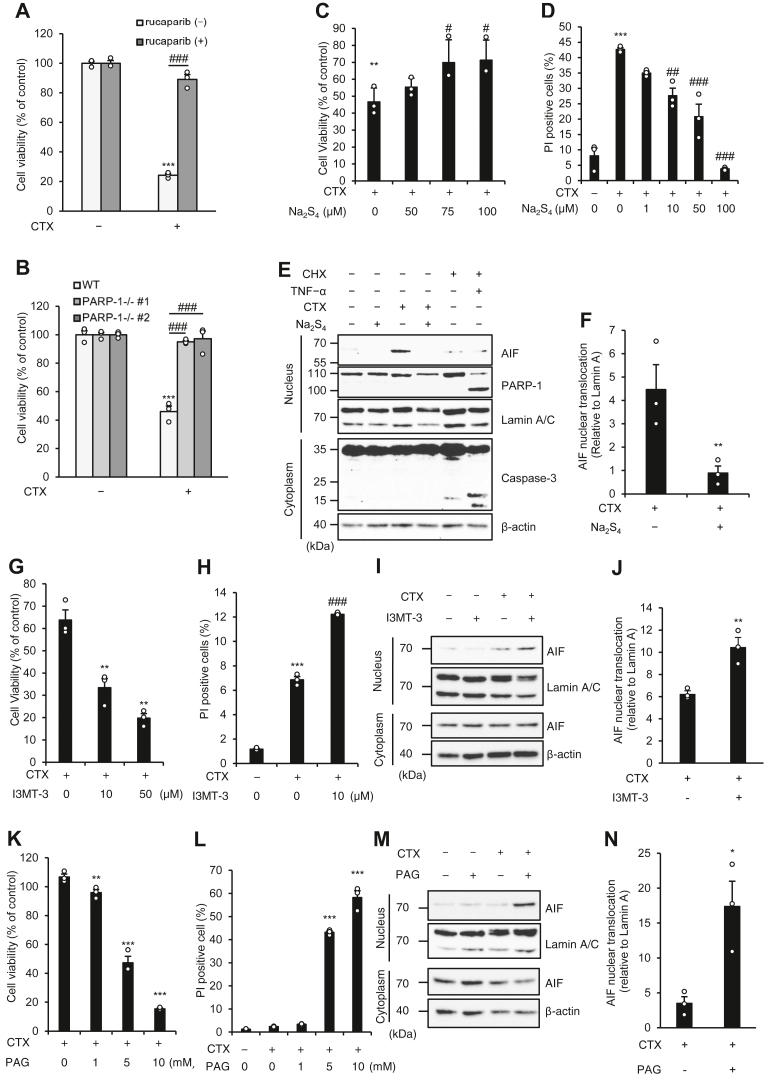
**RSS suppress oxidative stress–induced parthanatos.***A*, HT1080 cells were treated with CTX (1 mg/ml) and/or rucaparib (1 μM) for 42 h. Cell viability was determined by PMS/MTS assay. Data shown are the mean ± SEM (n = 3). Significant differences were determined by one-way ANOVA, followed by Tukey–Kramer test; ∗∗∗*p* < 0.001, (*versus* control cells), ###*p* < 0.001 (*versus* CTX 1 mg/ml, rucaparib 0 μM cells). *B*, HT1080 and PARP-1 KO HT1080 were treated with CTX (1 mg/ml) for 48 h. Cell viability was determined by PMS/MTS assay. Data shown are the mean ± SEM (n = 3). Significant differences were determined by one-way ANOVA, followed by Tukey–Kramer test; ∗∗∗*p* < 0.001, (*versus* WT control cells), ###*p* < 0.001 (*versus* WT CTX 1 mg/ml cells). *C*, HT1080 cells were treated with CTX (1 mg/ml) with indicated concentration of Na_2_S_4_ for 48 h. Cell viability was determined by PMS/MTS assay. Data shown are the mean ± SEM (n = 3). Statistical significance was tested using an unpaired Student’s *t* test; ∗∗*p* < 0.01, (*versus* control cells), #*p* < 0.05, (*versus* CTX 1 mg/ml, Na_2_S_4_ 0 μM cells). *D*, HT1080 cells were treated with CTX (1 mg/ml) with indicated concentration of Na_2_S_4_ for 48 h. Dead cells were labeled with PI for 15 min and analyzed by FACS. Data shown are the mean ± SEM (n = 3). Significant differences were determined by one-way ANOVA, followed by Tukey–Kramer test; ∗∗∗*p* < 0.001, (*versus* control cells), ##*p* < 0.01, ###*p* < 0.001, (*versus* CTX 1 mg/ml, Na_2_S_4_ 0 μM cells). *E*, HT1080 cells were treated with CTX (1 mg/ml) and/or Na_2_S_4_ (100 μM) for 36 h or CHX (10 μg/ml) and TNF-α (25 μg/ml) for 12 h. Cell lysates were subjected to immunoblotting with the indicated antibodies. *F*, nuclear AIF expressions were quantified using Image Lab software from Bio-Rad. Graphs depict the mean ± SEM of three independent experiments. Significant differences were determined by one-way ANOVA, followed by Tukey–Kramer test; ∗∗*p* < 0.01 (*versus* CTX 1 mg/ml, Na_2_S_4_ 0 μM cells). *G*, HT1080 cells were treated with CTX (1 mg/ml) with indicated concentration of I3MT-3 for 48 h. Cell viability was determined by PMS/MTS assay. Data shown are the mean ± SEM (n = 3). Significant differences were determined by one-way ANOVA, followed by Tukey–Kramer test; ∗∗*p* < 0.01, ∗∗∗ < 0.001 (*versus* CTX 1 mg/ml, I3MT-3 0 μM cells). *H*, HT1080 cells were treated with CTX (1 mg/ml) and/or I3MT-3 (10 μM) for 48 h. Dead cells were labeled with PI for 15 min and analyzed by FACS. Data shown are the mean ± SEM (n = 3). Significant differences were determined by one-way ANOVA, followed by Tukey–Kramer test; ∗∗∗*p* < 0.001, (*versus* control cells), ###*p* < 0.001, (*versus* CTX 1 mg/ml, I3MT-3 0 μM cells). *I*, HT1080 cells were treated with CTX (1 mg/ml) and/or I3MT-3 (10 μM) for 36 h. Cell lysates were subjected to immunoblotting with the indicated antibodies. *J*, nuclear AIF expressions were quantified using Image Lab software from Bio-Rad. Graphs depict the mean ± SEM of three independent experiments. Significant differences were determined by one-way ANOVA, followed by Tukey–Kramer test; ∗∗*p* < 0.01 (*versus* CTX 1 mg/ml, I3MT-3 0 μM cells). *K*, HT1080 cells were treated with CTX (1 mg/ml) with the indicated concentration of PAG for 24 h. Cell viability was determined by PMS/MTS assay. Data shown are the mean ± SEM (n = 3). Significant differences were determined by one-way ANOVA, followed by Tukey–Kramer test; ∗∗*p* < 0.051, ∗∗∗ < 0.001 (*versus* CTX 1 mg/ml, PAG 0 mM cells). *L*, HT1080 cells were treated with CTX (1 mg/ml) with the indicated concentration of PAG for 24 h. Dead cells were labeled with PI for 15 min and analyzed by FACS. Data shown are the mean ± SEM (n = 3). Significant differences were determined by one-way ANOVA, followed by Tukey–Kramer test; ∗∗∗*p* < 0.001, (*versus* CTX 1 mg/ml, PAG 0 mM cells). *M*, HT1080 cells were treated with CTX (1 mg/ml) and/or PAG (5 mM) for 36 h. Cell lysates were subjected to immunoblotting with the indicated antibodies. *N*, nuclear AIF expressions were quantified using Image Lab software from Bio-Rad. Graphs depict the mean ± SEM of three independent experiments. Significant differences were determined by one-way ANOVA, followed by Tukey–Kramer test; ∗*p* < 0.05 (*versus* CTX 1 mg/ml, PAG 0 mM cells). All data are representative of at least three independent experiments. AIF, apoptosis-inducing factor; CHX, cycloheximide; CTX, cefotaxime; FACS, fluorescence-activated cell sorting; MTS, 3-(4,5-dimethylthiazol-2-yl)-5-(3-carboxymethoxyphenyl)-2-(4-sulfophenyl)-2H-tetrazolium; PAG, DL-propargylglycine; PARP-1, poly (ADP-ribose) polymerase-1; PI, propidium iodide; PMS, phenazine methosulfate; RSS, reactive sulfur species.

We have previously demonstrated that cefotaxime (CTX), a third-generation cephalosporin antibiotic, clearly promotes mitochondrial ROS generation and particularly induces oxidative stress-induced parthanatos in human fibrosarcoma HT1080 cell line (13, 33, 34). As shown in Figure 1, *A* and *B*, reduced cell viability upon CTX treatment was significantly restored by the PARP-1 inhibitor rucaparib or PARP-1 KO, showing that CTX induces parthanatos in HT1080 cells. Interestingly, we observed that cotreatment with Na_2_S_4_ restores viability of CTX-treated HT1080 cells in a concentration-dependent manner (Fig. 1*C*). Propidium iodide (PI) staining assays revealed that Na_2_S_4_ reduces PI-positive (dead) cells even at low concentration (1 μM) (Fig. 1*D*). Moreover, consistent with these observations, Na_2_S_4_ clearly inhibited nuclear translocation of AIF that triggers large-scale DNA fragmentation (Fig. 1, *E* and *F*). Meanwhile, tumor necrosis factor-α in the presence of cycloheximide clearly promoted the cleavage of caspase-3 and PARP-1, while CTX failed to do so, further confirming that CTX induces parthanatos but not apoptosis (Fig. 1*E*). Collectively, these observations suggest that RSS produced by Na_2_S_4_ suppress CTX-induced parthanatos. We next examined the involvement of endogenously produced RSS in CTX-induced parthanatos. As shown in Figure 1, *G* and *H*, the loss of cell viability was enhanced, and the ratio of PI-positive cells was increased in the presence of I3MT-3 (HMPSNE), a potent and selective inhibitor of the RSS-producing enzyme 3-MST. In addition, I3MT-3 enhanced CTX-induced nuclear translocation of AIF (Fig. 1, *I* and *J*). Similar to I3MT-3, the CSE inhibitor DL-propargylglycine (PAG) significantly enhanced the loss of cell viability induced by CTX and increased the ratio of PI-positive cells accompanied by enhanced nuclear translocation of AIF (Fig. 1, *K*–*N*). Notably, inhibition of CSE strongly induced parthanatos even under conditions in which CTX alone failed to induce cell death (Fig. 1*L*). Therefore, endogenous RSS produced by CSE especially appear to contribute to the suppression of CTX-induced parthanatos.

### RSS suppress the ALIS formation

**Figure 2:**
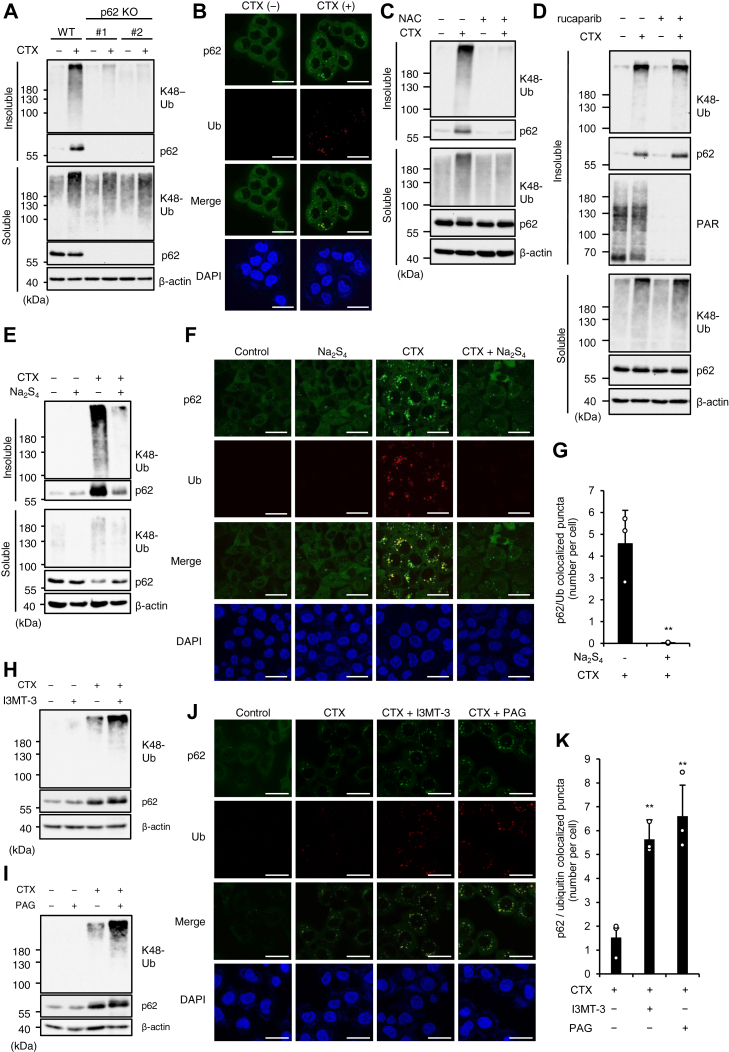
**RSS suppress the ALIS formation.***A*, HT1080 and p62 KO HT1080 cells were treated with CTX (1 mg/ml) for 24 h, and then the detergent-soluble and detergent-insoluble fractions were subjected to immunoblotting with the indicated antibodies. *B*, HT1080 cells were treated with CTX (1 mg/ml) for 24 h, then performed immunofluorescence staining with the indicated antibody, and 4′,6-diamidino-2-phenylindole (DAPI) nuclear staining. Scale bar represents 10 μm. *C*, HT1080 cells were treated with CTX (1 mg/ml) and/or NAC (2 mM) for 24 h, and then the detergent-soluble and detergent-insoluble fractions were subjected to immunoblotting with the indicated antibodies. *D*, HT1080 cells were treated with CTX (1 mg/ml) and/or rucaparib (1 μM) for 24 h, and then the detergent-soluble and detergent-insoluble fractions were subjected to immunoblotting with the indicated antibodies. *E*, HT1080 cells were treated with CTX (1 mg/ml) and/or Na_2_S_4_ (100 μM) for 24 h, and then the detergent-soluble and detergent-insoluble fractions were subjected to immunoblotting with the indicated antibodies. *F*, HT1080 cells were treated with CTX (1 mg/ml) and/or Na_2_S_4_ (100 μM) for 24 h, then performed immunofluorescence staining with the indicated antibody, and DAPI nuclear staining. Scale bar represents 10 μm. *G*, the number of p62 and ubiquitin-colocalized puncta were quantified using Image J. Data shown are the mean ± SEM (n = 3). Significant differences were determined by one-way ANOVA, followed by Tukey–Kramer test; ∗∗*p* < 0.01, (*versus* CTX 1 mg/ml, Na_2_S_4_ 0 μM cells). *H*, HT1080 cells were treated with CTX (1 mg/ml) and/or I3MT-3 (10 μM) for 24 h, and then whole cell lysates were subjected to immunoblotting with the indicated antibodies. *I*, HT1080 cells were treated with CTX (1 mg/ml) and/or PAG (5 mM) for 24 h, and then whole cell lysates were subjected to immunoblotting with the indicated antibodies. *J*, HT1080 cells were treated with the indicated reagents for 24 h, then performed immunofluorescence staining with the indicated antibody, and DAPI nuclear staining. CTX (1 mg/ml). I3MT-3 (10 μM). PAG (5 mM). Scale bar represents 10 μm. *K*, the number of p62 and ubiquitin-colocalized puncta were quantified using Image J. Data shown are the mean ± SEM (n = 3). Significant differences were determined by one-way ANOVA, followed by Tukey–Kramer test; ∗∗*p* < 0.01, (*versus* CTX 1 mg/ml, I3MT-3 0 μM PAG 0 mM cells). All data are representative of at least three independent experiments. ALIS, aggresome-like induced structure; CTX, cefotaxime; PAG, DL-propargylglycine; NAC, N-acetylcysteine; RSS, reactive sulfur species.

We next examined how RSS suppress CTX-induced parthanatos. Our previous study has demonstrated that oxidative stress-induced ALIS formation is required for CTX-induced parthanatos (13). As previously described, CTX-induced ALIS formation is biochemically shown as the accumulation of p62 and K48-linked polyubiquitinated proteins in the detergent-insoluble fraction, while p62-ALIS is microscopically observed as costained puncta of p62 and K48-linked ubiquitin (Fig. 2, *A* and *B*) (13). The accumulation of p62 and K48-linked polyubiquitinated proteins in the detergent-insoluble fraction was clearly abrogated by treatment with the antioxidant *N*-acetylcysteine or p62 KO as previously published data (13), confirming that CTX-induced ALIS formation depends on oxidative stress and p62 (Fig. 2, *A* and *C*). On the other hand, rucaparib failed to inhibit the accumulation of p62 and K48-linked polyubiquitinated proteins in the detergent-insoluble fraction, whereas the accumulation of poly-ADP–ribosylated proteins was clearly inhibited (Fig. 2*D*).

Interestingly, Na_2_S_4_ also suppressed the accumulation of p62 and K48-linked polyubiquitinated proteins in the detergent-insoluble fraction (Fig. 2*E*). Moreover, immunofluorescent puncta of p62 and ubiquitinated proteins were clearly reduced by Na_2_S_4_ treatment (Fig. 2, *F* and *G*). On the other hand, both I3MT-3 and PAG enhanced CTX-induced ALIS formation and led to increase the intensity of the costained p62 and K48-linked ubiquitin puncta (Fig. 2, *H*–*K*). Thus, these results suggest that RSS suppress CTX-induced parthanatos by preventing the p62-based ALIS formation.

### RSS disaggregate the ALIS by inducing HSP70

**Figure 3:**
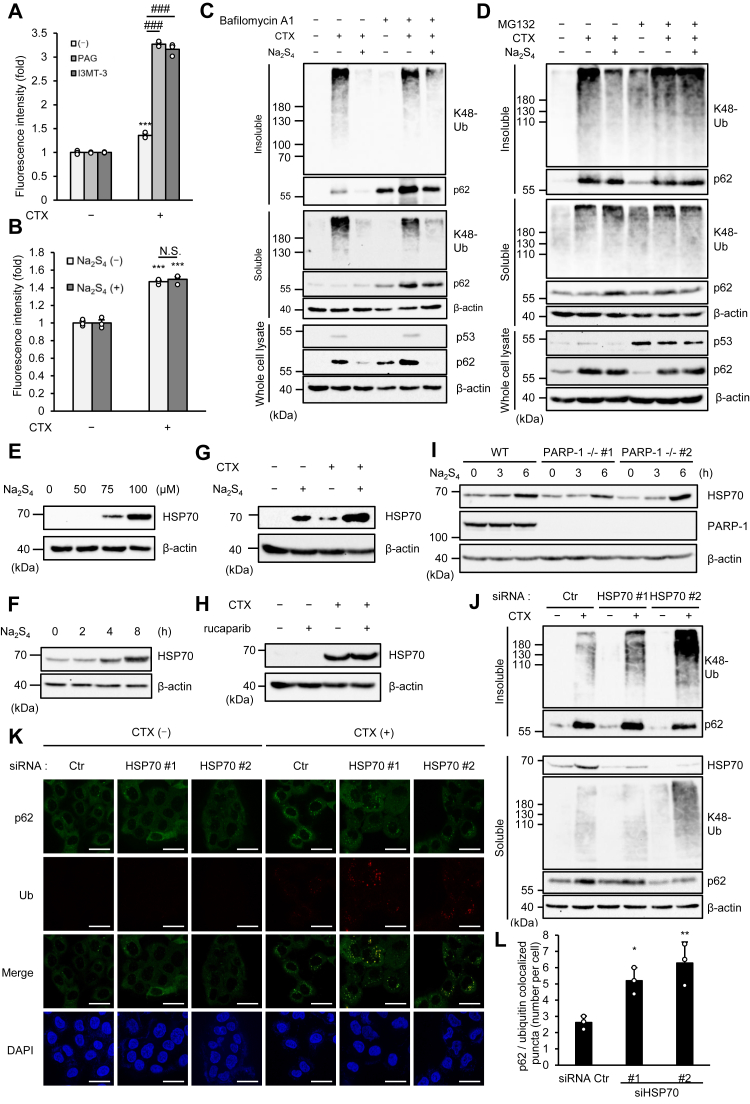
**RSS disaggregate the ALIS by inducing HSP70.***A*, HT1080 cells were treated with the indicated reagents for 24 h and then incubated with 10 μM 2′,7′-dichlorodihydrofluorescein diacetate (DCFH-DA). Quantification of ROS was calculated by detecting the fluorescence intensity of DCFH-DA. CTX (1 mg/ml). PAG (5 mM). I3MT-3 (20 μM). Data shown are the mean ± SEM (n = 3). Significant differences were determined by one-way ANOVA, followed by Tukey–Kramer test; ∗∗∗*p* < 0.001, (*versus* control cells), ###*p* < 0.001, (*versus* CTX 1 mg/ml, PAG 0 mM, I3MT-3 0 μM cells). *B*, HT1080 cells were treated with CTX (1 mg/ml) and/or Na_2_S_4_ (100 μM) for 24 h and then incubated with 10 μM DCFH-DA. Quantification of ROS was calculated by detecting the fluorescence intensity of DCFH-DA. Data shown are the mean ± SEM (n = 3). Significant differences were determined by one-way ANOVA, followed by Tukey–Kramer test; ∗∗∗*p* < 0.001, (*versus* control cells), N.S. *p* > 0.05 (*versus* CTX 1 mg/ml Na_2_S_4_ 0 μM cells). *C*, HT1080 cells were treated with the indicated reagents for 24 h, and then the detergent-soluble and detergent-insoluble fractions and whole cell lysate were subjected to immunoblotting with the indicated antibodies. Bafilomycin A1 (5 nM). CTX (1 mg/ml). Na_2_S_4_ (100 μM). *D*, HT1080 cells were treated with CTX (1 mg/ml) for 20 h and then treated with MG132 (10 μM) and/or Na_2_S_4_ (100 μM) for 4 h. The detergent-soluble and detergent-insoluble fractions and whole cell lysate were subjected to immunoblotting with the indicated antibodies. *E*, HT1080 cells were treated with the indicated concentration of Na_2_S_4_ for 24 h, and then whole cell lysates were subjected to immunoblotting with the indicated antibodies. *F*, HT1080 cells were treated with Na_2_S_4_ (100 μM) for indicated period, and then whole cell lysates were subjected to immunoblotting with the indicated antibodies. *G*, HT1080 cells were treated with CTX (1 mg/ml) and Na_2_S_4_ (100 μM) for 18 h, and then whole cell lysates were subjected to immunoblotting with the indicated antibodies. *H*, HT1080 cells were treated with CTX (1 mg/ml) and/or rucaparib (1 μM) for 24 h, and then whole cell lysate were subjected to immunoblotting with the indicated antibodies. *I*, HT1080 and PARP-1 KO cells were treated with Na_2_S_4_ (100 μM) for indicated period, and then whole cell lysates were subjected to immunoblotting with the indicated antibodies. *J*, HT1080 cells were transfected with siRNA for negative control or HSP70 (HSP70 #1 or HSP70 #2). After 24 h, the cells were treated with CTX (1 mg/ml) for 24 h, and then the detergent-soluble and detergent-insoluble fractions were subjected to immunoblotting with the indicated antibodies. *K*, HT1080 cells were transfected with siRNA for negative control or HSP70 (HSP70 #1 or HSP70 #2). After 24 h, the cells were treated with CTX (1 mg/ml) for 24 h, then performed immunofluorescence staining with the indicated antibody, and 4′,6-diamidino-2-phenylindole (DAPI) nuclear staining. Scale bar represents 10 μm. *L*, the number of p62 and ubiquitin-colocalized puncta were quantified using Image J. Data shown are the mean ± SEM (n = 3). Significant differences were determined by one-way ANOVA, followed by Tukey–Kramer test; ∗*p* < 0.05, ∗∗*p* < 0.01, (*versus* siRNA Ctr, CTX 1 mg/ml cells). All data are representative of at least three independent experiments. ALIS, aggresome-like induced structure; CTX, cefotaxime; HSP, heat shock protein; PAG, DL-propargylglycine; PARP-1, poly (ADP-ribose) polymerase-1; ROS, reactive oxygen species; RSS, reactive sulfur species.

We therefore explored mechanisms by which RSS prevent the p62-based ALIS formation. As previous studies have demonstrated that RSS mitigate oxidative stress (35), both the inhibitors of 3-MST and CSE clearly enhanced CTX-induced ROS generation (Fig. 3*A*). However, interestingly, Na_2_S_4_ did not affect CTX-induced ROS generation (Fig. 3*B*). These results therefore suggest that additionally generated RSS by the Na_2_S_4_ treatment do not contribute to ROS elimination and prevent the p62-based ALIS formation by other mechanisms. We then speculated that RSS negatively regulate the p62-based ALIS formation at protein levels. In general, protein aggregates are removed by the autophagic or the ubiquitin-proteasome system. To test the possibility that RSS suppress the ALIS formation by activating these systems, the effects of Na_2_S_4_ on the ALIS formation were evaluated in the presence of the autophagy inhibitors bafilomycin A1 or the proteasome inhibitor MG132. As shown in Figure 3*C*, bafilomycin A1 elevated the expression levels of p62 in both detergent-soluble and detergent-insoluble fractions due to a lack of the lysosomal degradation of p62. Meanwhile, MG132 elevated the expression levels of p53 that is constitutively degraded by the proteasome (Fig. 3*D*). Interestingly, Na_2_S_4_ prevented the ALIS formation even in the presence of bafilomycin A1, whereas Na_2_S_4_ failed to do so in the presence of MG132, suggesting that the ubiquitin-proteasome but not the autophagic system is involved in the effects of Na_2_S_4_ (Fig. 3, *C* and *D*). Accumulating evidence suggests that molecular chaperones mediate proteasomal degradation of misfolded proteins by delivering them to the 26S proteasomes (36). We therefore speculated the involvement of molecular chaperones in disaggregation of the ALIS. In particular, it is known that the molecular chaperone HSP70 is closely associated with oxidative stress, and we have previously demonstrated that CTX-induced ROS generation upregulates HSP70 (34, 37). Interestingly, we found that Na_2_S_4_ clearly elevates the expression of HSP70 protein in a time- and concentration-dependent fashion (Fig. 3, *E* and *F*). Moreover, the upregulation of HSP70 by Na_2_S_4_ was more potent than that by CTX, and Na_2_S_4_ and CTX cotreatment additively upregulated HSP70 (Fig. 3*G*). Recent evidence has shown that PARP-1 is involved in the regulation of HSPs, including HSP70, which prompted us to test whether PARP-1 is required for the upregulation of HSP70 by Na_2_S_4_ (38, 39). However, as shown in Figure 3, *H* and *I*, the upregulation of HSP70 by Na_2_S_4_ was not affected by pharmacological inhibition or KO of PARP-1, suggesting that contribution of PARP-1 to the upregulation of HSP70 by Na_2_S_4_ is relatively small in this context. We next examined whether HSP70 disaggregates the p62-based ALIS. Immunoblot analysis revealed that knockdown of HSP70 clearly increased the K48-linked polyubiquitinated proteins in the insoluble fraction (Fig. 3*J*). Moreover, K48-linked ubiquitin puncta were increased in HSP70 knockdown cells (Fig. 3, *K* and *L*). These findings suggest that HSP70 inhibits the accumulation of the K48-linked polyubiquitinated proteins in the p62-based ALIS and further suggest the possibility that RSS disaggregates the ALIS by inducing HSP70.

### RSS activate HSF1 by promoting its dissociation from HSP90

**Figure 4:**
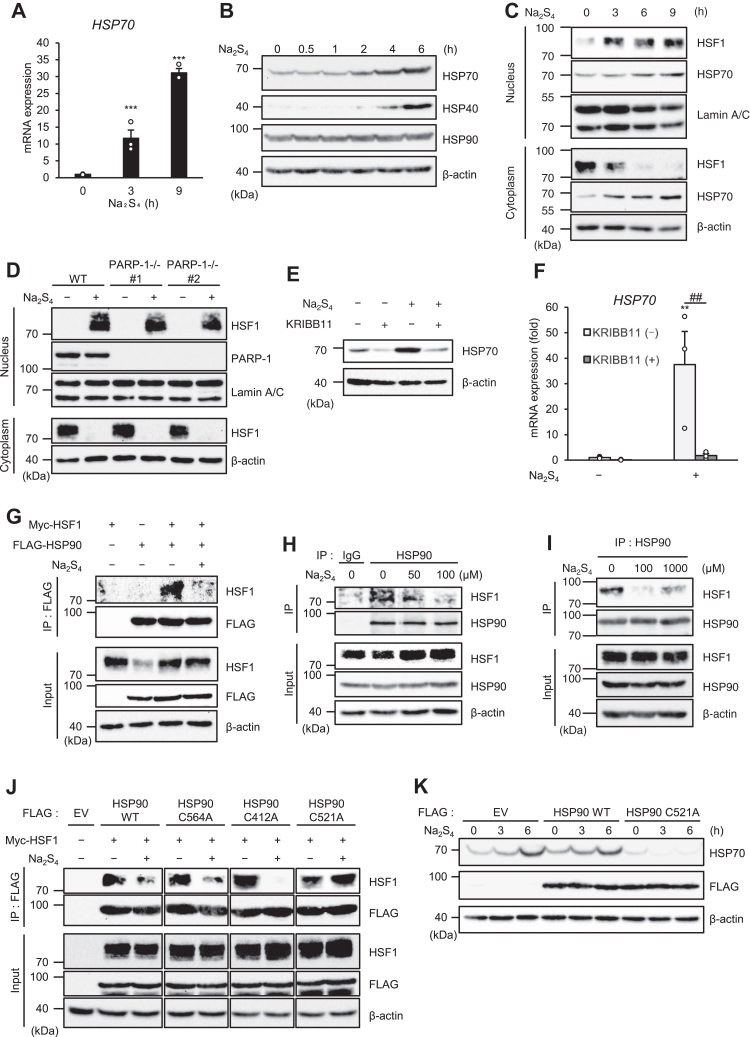
**RSS activate HSF1 by promoting its dissociation from HSP90.***A*, HT1080 cells were treated with the Na_2_S_4_ (100 μM) for indicated period, and then the mRNA levels were measured by quantitative real-time PCR. Data shown are the mean ± SEM (n = 3). Statistical significance was tested using an unpaired Student’s *t* test; ∗∗∗*p* < 0.001, (*versus* control cells). *B*, HT1080 cells were treated with the Na_2_S_4_ (100 μM) for the indicated period. Cell lysates were subjected to immunoblotting with the indicated antibodies. *C*, HT1080 cells were treated with the Na_2_S_4_ (100 μM) for the indicated period. Cell lysates were subjected to immunoblotting with the indicated antibodies. *D*, HT1080 and PARP-1 KO cells were treated with the Na_2_S_4_ (100 μM) for 6 h. Cell lysates were subjected to immunoblotting with the indicated antibodies. *E*, HT1080 cells were treated with the Na_2_S_4_ (100 μM) and KRIBB11 (10 μM) for 12 h, and then cell lysates were subjected to immunoblotting with the indicated antibodies. *F*, HT1080 cells were treated with the Na_2_S_4_ (100 μM) and KRIBB11 (10 μM) for 12 h, and then the mRNA levels were measured by quantitative real-time PCR. Data shown are the mean ± SEM (n = 3). Significant differences were determined by one-way ANOVA, followed by Tukey–Kramer test; ∗∗*p* < 0.01, (*versus* control cells), ##*p* < 0.01 (*versus* Na_2_S_4_ 100 μM, KRIBB11 0 μM cells). *G*, HT1080 cells were transfected with FLAG-HSP90 and/or Myc-HSF1 plasmid for 24 h and treated with Na_2_S_4_ (100 μM) for 4 h, then immunoprecipitated anti-FLAG-tagged agarose beads, and subjected to immunoblotting with the indicated antibodies. *H*, HT1080 cells were treated with indicated concentration of Na_2_S_4_ for 4 h, then immunoprecipitated protein G-Sepharose beads with the indicated antibodies, and subjected to immunoblotting with the indicated antibodies. *I*, HSP90 was immunoprecipitated using anti-HSP90 antibody with protein G beads. Beads were washed four times with PBS and then treated with Na_2_S_4_ (100, 1000 μM) for 1 h. After reaction, beads were washed four times with PBS and subjected to immunoblotting with the indicated antibodies. *J*, HT1080 cells were transfected with FLAG-HSP90 (WT/C412A/C564A/C521A) and Myc-HSF1 plasmid for 24 h and treated with Na_2_S_4_ (100 μM) for 4 h, then immunoprecipitated anti-FLAG-tagged agarose beads, and subjected to immunoblotting with the indicated antibodies. *K*, HT1080 cells were transfected with FLAG-Empty or FLAG-HSP90 (WT/C521A) plasmid for 24 h and treated with Na_2_S_4_ (100 μM) for indicated periods. Cell lysates were subjected to immunoblotting with the indicated antibodies. All data are representative of at least three independent experiments. HSF, heat shock factor; HSP, heat shock protein; PARP-1, poly (ADP-ribose) polymerase-1; RSS, reactive sulfur species.

**Figure 5:**
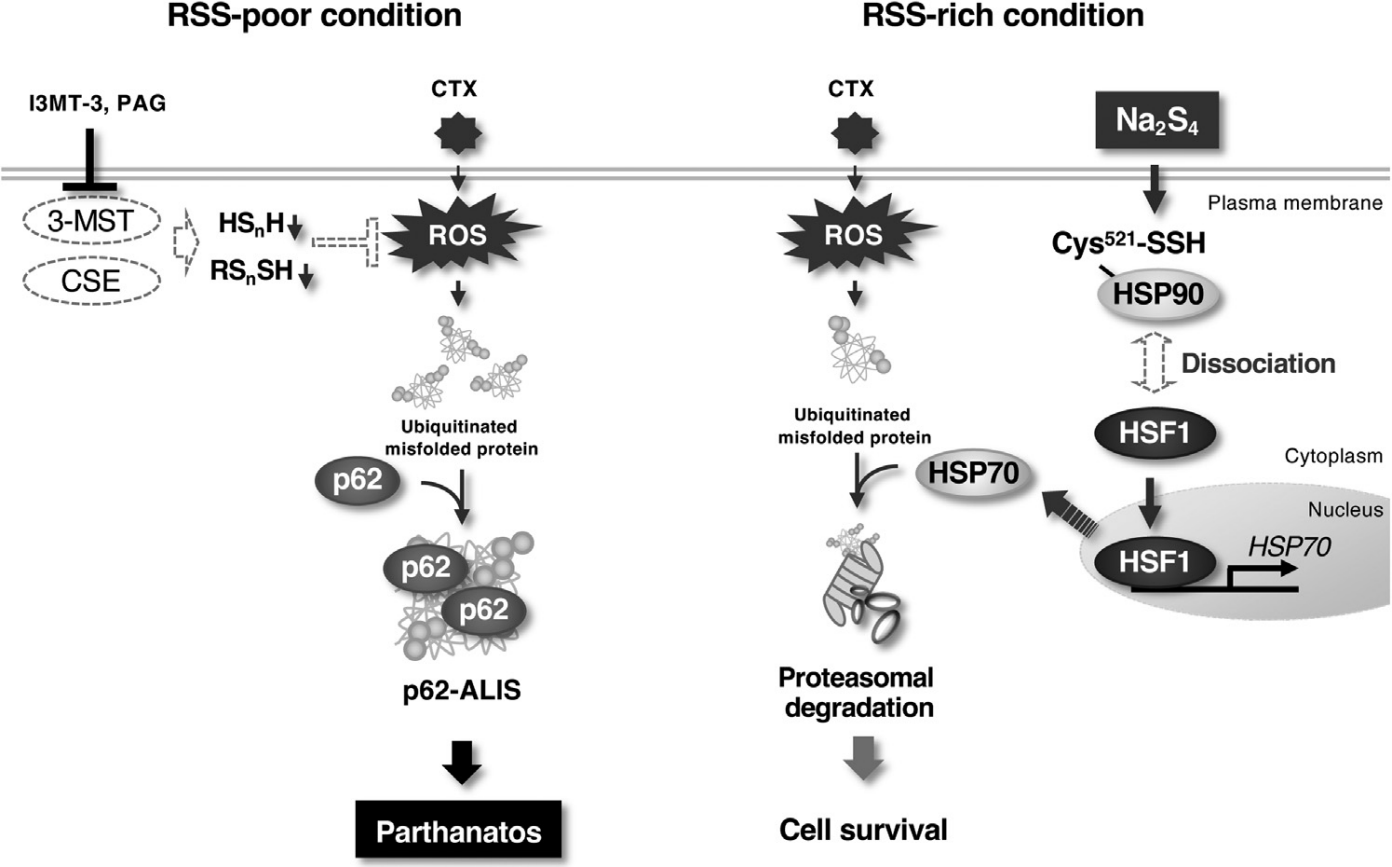
**Schematic model to explain our study.** Loss of endogenous RSS promotes ROS-dependent accumulation of the denatured proteins, possibly due to failure of ROS elimination. Thereafter, the accumulation of the denatured proteins stimulates the formation of the p62-ALIS, which triggers parthanatos. On the other hand, abundant RSS supply contributes to the persulfidation of HSP90 on Cys521 and thereby dissociation of HSP90 from HSF1, rather than ROS elimination. As a result, HSF1 is activated and translocated to the nucleus and then HSP70 is strongly upregulated. Upregulated HSP70 leads to the proteasomal degradation of denatured proteins and thereby prevents the p62-ALIS formation and subsequent parthanatos. Further considerations are described in the Discussion section. ALIS, aggresome-like induced structure; HSF, heat shock factor; HSP, heat shock protein; ROS, reactive oxygen species; RSS, reactive sulfur species.

We next investigated mechanisms by which RSS upregulate HSP70. Quantitative real-time PCR analysis revealed that Na_2_S_4_ strongly induces mRNA levels of HSP70 (Fig. 4*A*). Moreover, in addition to HSP70, Na_2_S_4_ induces HSP40 but not HSP90 at protein levels (Fig. 4*B*). These observations raise the possibility that Na_2_S_4_ acts on HSF1, a master regulator of HSPs. As expected, Na_2_S_4_ promoted nuclear translocation of HSF1, a transcriptional regulator of HSP70, which was not inhibited by PARP-1 KO (Fig. 4, *C* and *D*). Interestingly, KRIBB11, recently identified as a HSF1 inhibitor (40), strongly inhibited Na_2_S_4_-induced HSP70 upregulation at both protein and mRNA levels (Fig. 4, *E* and *F*). Therefore, these results suggest that RSS upregulate HSP70 at mRNA levels through the HSF1-dependent mechanism. We next explored the mechanism by which RSS activate HSF1. Immunoprecipitation experiments revealed that Na_2_S_4_ promotes the dissociation of HSF1 from HSP90, which allows the nuclear translocation of HSF1 (Fig. 4*G*). The dissociation was confirmed by the coimmunoprecipitation assay of endogenous proteins (Fig. 4*H*). Moreover, the dissociation of HSF1 from HSP90 was observed by the *in vitro* treatment of Na_2_S_4_ (Fig. 4*I*). These observations raise the possibility that RSS directly act on the HSF1–HSP90 complex and execute their dissociation. Of note, recent reports have demonstrated that the chemical modifications of Cys 412, 521, and 564 in HSP90 allow the conformational changes of HSP90 protein (41, 42, 43). Therefore, we speculated that RSS promote the dissociation of the HSF1–HSP90 complex through the persulfidation of these cysteines in HSP90. As shown in Figure 4*J*, only HSP90 C521A mutant in which cysteine (C) 521 was substituted by alanine (A) failed to dissociate from HSF1 even in the presence of RSS. Moreover, the HSP70 induction induced by Na_2_S_4_ was strongly inhibited by the expression of HSP90 C521A mutant (Fig. 4*K*). Therefore, HSP90 C521A mutant appears to act as a dominant-negative mutant. Taken together, these findings further suggest that the persulfidation of HSP90 at Cys521 by RSS triggers the conformational changes of HSP90, leading to the dissociation of HSP1. Based on these results, we propose a model in which RSS prevent parthanatos mediated by the ALIS (Fig. 5).

## Discussion

In this study, we demonstrated that RSS suppress the ALIS formation and subsequent parthanatos by upregulating HSP70. Unexpectedly, the suppression of the ALIS formation by RSS donors was shown to depend on proteasomal degradation rather than removal of ROS. The proteasomal degradation of aggregating or hydrophobic ubiquitinated proteins is known to be mediated by the chaperone proteins such as HSP40 and HSP70 (30). Interestingly, we found that RSS promote the transcription of HSP70 by promoting the dissociation of HSF1 from its inhibitor HSP90. Although mechanisms by which HSP90 sense stresses remain unclear, chemical or oxidative modifications of HSP90 has been reported to be involved in the HSF1 activation (41, 42). In particular, modification of cysteine (Cys) 412, 521, 564 in HSP90 by the electrophiles is required for the HSF1 activation, indicating that the cysteine residue of HSP90 acts as a redox sensor to regulate the HSF1 activity (43). Of note, Na_2_S_4_ promoted the dissociation of the HSP90–HSF1 complex not only in cells but also *in vitro* (Fig. 4*H*). These mechanistic findings suggest that RSS directly target and modify the cysteine residue of HSP90 to regulate the HSF1 activation. Accumulating evidence has shown that RSS alter the activity of proteins or GSH by persulfidation or polysulfidation of its cysteine residues (20). Moreover, recent reports have demonstrated that some of the cysteine residues in HSP90, PTEN, PTP1B, and keap1 exist as cysteine persulfides, which serves to avoid irreversible oxidation of the cysteine residues (19). It has been reported that chemical modification of the cysteine residue of HSP90 induces its conformational change and promotes dissociation from HSF1 (41, 42, 43). Interestingly, in this study, only mutation of Cys521 inhibited the dissociation of HSF1 by Na_2_S_4_. Therefore, RSS appear to induce the HSF1 activation *via* persulfidation of Cys521 of HSP90.

On the other hand, we focused on the RSS-producing enzyme 3-MST and CSE and examined their effects on CTX-induced parthanatos. Interestingly, the specific inhibitor of both 3-MST and CSE clearly promoted CTX-induced ROS accumulation, whereas the Na_2_S_4_ treatment failed to so (Fig. 3, *A* and *B*). These observations suggest that RSS produced by 3-MST and CSE but not Na_2_S_4_ has an ability to eliminate ROS. Although further studies are required to explain this difference, distinct types of RSS may be produced by 3-MST, CSE, and Na_2_S_4_. 3-MST and CSE produce RSS *via* both hydrogen sulfide (H_2_S) and H_2_Sn, while Na_2_S_4_ basically produces RSS *via* only H_2_S_4_ (44, 45). Thus, it is likely that RSS produced by H_2_S mainly contributes to the ROS elimination, while RSS produced by H_2_S_4_ contributes to the protein quality control through the HSP70 induction.

Overactivation of PARP-1 and parthanatos has been implicated in the development of various neurodegenerative diseases, and therefore suppression of parthanatos is thought to be a potential therapeutic strategy for neurodegenerative diseases (1). Interestingly, protein persulfidation is also known to be associated with neurodegenerative diseases. For example, persulfidation of parkin promotes proteasomal degradation of denatured proteins by enhancing its E3 activity and suppresses Parkinson's disease, while persulfidation of glycogen synthase kinase 3β suppresses Alzheimer's disease by downregulating tau protein aggregation (46, 47). Meanwhile, multiple reports have shown that the HSF1 activation suppresses protein aggregation that causes neurodegenerative diseases, suggesting the possibility that the HSF1 activation caused by RSS is involved in the suppression of neurodegenerative diseases (31). Moreover, CSE is downregulated in neurodegenerative diseases such as Huntington's disease and is known to lead to the exacerbation of the disease (48). Thus, our results that the RSS donor clearly prevents the ALIS formation and subsequent parthanatos may open the possibility of RSS as a therapeutic target for neurodegenerative diseases.

## Experimental procedures

### Reagents and plasmids

All reagents were obtained from commercial sources; CTX, sodium tetrasulfide (Na_2_S_4_), N-acetylcysteine, 2′,7′-dichlorodihydrofluorescein diacetate (WAKO), rucaparib, KRIBB11 (Selleck), I3MT-3 (MedChemExpress), PAG (Sigma-Aldrich). The antibodies used were against FLAG (Sigma), p62 (MBL), AIF, K48-linked ubiquitin, poly/mono-ADP Ribose (Cell Signaling Technology), β-actin, PARP-1, Lamin A/C, HSP70, HSF1 (Santa cruz). Complementary DNAs (cDNAs) encoding human HSP90AB1 was obtained by performing PCR and was inserted into pcDNA3 with Flag tag plasmid as described previously (49). Plasmid transfection was performed using PEI “Max” (Polysciences), according to the manufacturer’s instructions. siRNA targeting human HSP70 was obtained from GeneDesign (50). HT1080 cells were transfected with 10 nM nontargeting siRNA pool (Dharmacon) as control or HSP70 siRNAs using Lipofectamine RNAiMAX Transfection Reagent (Invitrogen), according to the manufacturer’s instructions. HSP70 siRNA sequences were 5′- CACGGCAAGGUGGAGAUCA′ (HSP70 #1) and 5′- UGCACCUUGGGCUUGUCUCCGUCGU′ (HSP70 #2).

### Cell lines

HT1080 cells were from Japanese Collection of Research Bioresources Cell Bank (51). HT1080 cells were grown in Dulbecco's Modified Eagle Medium , 10% heat-inactivated fetal bovine serum, and 1% penicillin-streptomycin solution, at 37 °C under a 5% CO_2_ atmosphere.

### Colorimetric cell viability assay

Cell viability assay was performed as described previously (52). Cells were seeded on 96-well plates at a density of 1 × 10^4^/well. After indicated stimulation or treatment, cell viability was determined using CellTiter 96 Cell Proliferation Assay (Promega), according to the manufacturer's protocol. The absorbance was read at 492 nm using a microplate reader. Data are normalized to control (100%) without stimulus, unless noted otherwise.

### Immunoblot

Cells were seeded on 12-well plates at a density of 2 × 10^5^/well. After indicated stimulation or treatment, cells were suspended in 1% Triton X-100 buffer [20 mM Tris–HCl (pH 7.4), 150 mM NaCl, 1% Triton-X100, 10% glycerol, and 1% protease inhibitor cocktails (Nacalai Tesque)] for 15 min. Cell lysates were centrifuged at 4 °C at 15,000 rpm for 15 min, and then the supernatants were collected as detergent-soluble fraction. After washing two times with 1% Triton X-100 buffer, the pellets were resuspended in Laemmli sample buffer [62.5 mM Tris–HCl (pH 6.8), 2% SDS, 10 % glycerol] for 15 min. The lysates were centrifuged at 4 °C at 15,000 rpm for 5 min, and then the supernatants were collected as detergent-insoluble fraction. For preparation of whole cell lysates, cells were suspended in Laemmli sample buffer for 15 min, and then the lysates were centrifuged at 4 °C at 15,000 rpm for 5 min. The supernatants were collected as whole cell lysates. Protein concentration was determined using DC protein assay kit (Bio-Lab) according to the manufacturer’s instructions. Both the detergent-soluble and detergent-insoluble fractions and the whole cell lysate were subjected to immunoblot analysis as previously described (53).

### Immunoprecipitation

Immunoprecipitation was carried out with a modified procedure as previously described (54). After indicated stimulation or treatment, cells were lysed in ice-cold lysis buffer A [10 mM Hepes (pH 7.5), 10 mM KCl, 0.1 mM EGTA, 0.1 mM EDTA, 1 mM DTT, and 1% protease inhibitor cocktails (Nacalai Tesque)] for 15 min. Cell lysates were added 1% NP-40 and then centrifuged at 4 °C at 15,000 rpm for 15 min. The cell extracts were immunoprecipitated with 20 μl anti-FLAG–tagged agarose beads or protein G-Sepharose beads (Amersham Biosciences) for 2 h at 4 °C with the indicated antibodies. The beads were washed four times with the same buffer before analysis by SDS-PAGE.

### Semi *in vitro* dissociation assay

HT1080 cells (1 × 10ˆ6 cells/one sample) were lysed in buffer A plus 0.2% Nonidet P-40 and 1 mm DTT as described above, and HSP90–HSF1 complex was immunoprecipitated using anti-HSP90 antibody with protein G-Sepharose beads. Beads were washed four times with PBS and then treated with indicated reagent. After reaction, beads were washed four times with PBS and subjected to immunoblotting with the indicated antibodies.

### Fluorescence-activated cell sorting analysis

Fluorescence-activated cell sorting analysis was performed as described previously (55). Cells were seeded on 12-well plates at a density of 2 × 10^5^/well. For measurement of ROS generation, HT1080 cells were treated with indicated reagents and then incubated with 10 μM 2′,7′-dichlorodihydrofluorescein diacetate (Wako) for 30 min. The cells were detached from the culture dish by trypsin treatment and dispersed by pipetting as mild as possible to avoid mechanical damages to the cells. Fluorescence intensity was measured by flow cytometry with the excitation wavelength at 488 nm and the emission wavelength at 580 nm. For PI staining, HT1080 cells were treated with CTX and Na_2_S_4_, I3MT-3, or PAG and then labeled with 2 μg/ml PI for 15 min. Fluorescent cells were detected by CytoFLEX (Beckman Coulter), and dead cells were analyzed by using CytExpert (Beckman Coulter).

### Generation of KO cell lines

KO cells were generated using the CRISPR/Cas9 system (56). Guide RNAs were designed to target a region exon 3 of p62 gene (5′-AGACTACGACTTGTGTAGCG-3′) and PARP-1 gene (5′-GAGTCGAGTACGCCAAGAGC-3′) using CRISPRdirect. Guide RNA–encoding oligonucleotide was cloned into lenti- CRISPRv2 plasmid (addgene), and KO cells were established and characterized as previously described (13). To determine the mutations of p62 and PARP-1 in cloned cells, genomic sequence around the target region was analyzed by PCR-direct sequencing using extracted DNA from each clone as a template and the following primers:

For determination of p62 mutation; forward: 5′-GAGGACTTTAGGGGGTCCCA-3′, reverse: 5′-AGGAATTAGCAGAGCGGCAG-3′. For determination of PARP-1 mutation; forward: 5′-GCATCAGCAATCTATCAG-3′, reverse: 5′-CTTCCCGGACACAGTTAA-3′.

### Immunofluorescence staining

HT1080 cells were seeded on 12-well plates at a density of 2 × 10^5^/well. Cells were fixed with 3.7% formaldehyde, permeabilized with 0.5% Triton X-100, blocked with 3% bovine serum albumin-PBS, and incubated with primary antibodies (anti-ubiquitin) overnight at 4 °C, followed by incubation with secondary antibodies (goat anti-mouse Alexa Fluor 555, Invitrogen) for 1 h at room temperature. The immunostained samples were enclosed with Fluoro-KEEPER Antifade Reagent, Non-Hardening Type with DAPI (Nakalai), and observed using a Zeiss LSM800 laser confocal microscope.

### Nuclear extraction

Nuclear extraction was performed as described previously (57). Cells were seeded on 6-well plates at a density of 4 × 10^5^/well. After indicated stimulation or treatment, cells were lysed in ice-cold lysis buffer containing 10 mM Hepes (pH 7.5), 10 mM KCl, 0.1 mM EGTA, 0.1 mM EDTA, 1 mM DTT, and 1% protease inhibitor cocktails (Nacalai Tesque) for 15 min. Cell lysates were added 1% NP-40 and then centrifuged at 4 °C at 2500 rpm for 3 min. After the supernatants containing cytoplasmic fraction were removed, the pellets were suspended in ice-cold lysis buffer containing 20 mM Hepes (pH 7.5), 400 mM NaCl, 1 mM EGTA, 1 mM DTT, and 1% protease inhibitor cocktails for 15 min vortexed every 5 min. Cell lysates were then centrifuged at 4 °C at 15,000 rpm for 15 min, and then the supernatants were collected as nuclear fraction.

### Quantitative real-time PCR

HT1080 cells were seeded on 12-well plates at a density of 2 × 10^5^/well. After indicated stimulation or treatment, cells were suspended in Sepasol-RNA I Super G (Nacalai Tesque) for 15 min, and Chloroform:isoamyl alcohol (24:1, v/v) was added. Thereafter, samples were vortexed and centrifuged at 4 °C at 15,000 rpm for 15 min. In this process, DNA is fractionated into hydrophobic fraction, and RNA is fractionated into hydrophilic fraction. RNA was precipitated by adding 2-propanol to the hydrophilic fraction, allowing to stand for 15 min, and then centrifuging at 4 °C at 15,000 rpm for 15 min. The RNA pellets were washed twice with 70% ethanol, air-dried, and dissolved in RNase free water. RNA purity was confirmed by Nanodrop ND-1000 (Thermo Fisher Scientific). Total RNA was reverse transcribed using High Capacity cDNA Reverse Transcription Kit (Applied Biosystems) according to the manufacturer’s instructions. Template cDNA was amplified by quantitative real-time PCR as described previously (58). Primers used for quantitative real-time PCR are 5′- CGTGCTCATCTTTGACCTG -3′ and 5′- TGTTTTCTCTTGAACTCCTCCAC -3′ for human *HSP70*, 5′-AACAGCCTCAAGATCATCAGC -3′ and 5′-GGATGATGTTCTGGAGAGCC -3′ for human *GAPDH*. Each gene expression levels were normalized to that of *GAPDH*.

### Statistical analysis

The value was expressed as the mean ± SEM using Prism software (GraphPad; https://www.graphpad.com/features). All experiments were repeated at least three independent times. Two groups were compared using student’s *t* test. Multiple group comparisons were conducted using the one-way ANOVA followed by the Tukey–Kramer test using Prism software (GraphPad). Data were considered significant when ∗*p* < 0.05, ∗∗*p* < 0.01, ∗∗∗*p* < 0.001.

## Data availability

The data presented in this study are available in article.

## Conflict of interest

The authors declare no conflict of interest.
